# A process evaluation plan for assessing a complex community-based maternal health intervention in Ogun State, Nigeria

**DOI:** 10.1186/s12913-017-2124-4

**Published:** 2017-03-28

**Authors:** Sumedha Sharma, Olalekan O. Adetoro, Marianne Vidler, Sharla Drebit, Beth A. Payne, David O. Akeju, Akinmade Adepoju, Ebunoluwa Jaiyesimi, John Sotunsa, Zulfiqar A. Bhutta, Laura A. Magee, Peter von Dadelszen, Olukayode Dada

**Affiliations:** 1 Department of Obstetrics and Gynaecology and Child & Family Research Institute, University of British Columbia, Vancouver, Canada; 2 Centre for Research in Reproductive Health, Olabisi Onabanjo University Teaching Hospital, Sagamu, Nigeria; 3 University of Lagos, Lagos, Nigeria; 4 Babcock University, Sagamu, Nigeria; 5 Centre for Global Child Health, The Hospital for Sick Children, Toronto, Canada; 6 Centre of Excellence in Women and Child Health, Aga Khan University, Karachi, Pakistan; 7 Institute of Cardiovascular and Cell Sciences, St George’s, University of London, London, UK; 8 Department of Obstetrics and Gynaecology, St George’s University Hospitals NHS Foundation Trust, London, UK

**Keywords:** Process evaluation, Implementation research, Nigeria, Ogun, Maternal health, Complex interventions

## Abstract

**Background:**

Despite increased investment in community-level maternal health interventions, process evaluations of such interventions are uncommon, and can be instrumental in understanding mediating factors leading to outcomes. In Nigeria, where an unacceptably number of maternal deaths occur (maternal mortality ratio of 814/100,000 livebirths), the Community Level Interventions for Pre-eclampsia (CLIP) study (NCT01911494) aimed to reduce maternal and neonatal mortality and morbidity with a complex intervention of five interrelated components. Building from previous frameworks, we illustrate a methodology to evaluate implementation processes of the complex CLIP intervention, assess mechanisms of impact and identify emerging unintended causal pathways.

**Methods:**

The study was conducted from 2013–2016 in five Local Government Areas in Ogun State, Nigeria. A six-step approach was developed to evaluate key constructs of context (external factors related to intervention), implementation (fidelity, dose, reach, and adaption) and mechanisms of impact (unintended outcomes and mediating pathways). The steps are: 1) describing the intervention by a logic model, 2) defining acceptable delivery, 3) formulating questions, 4) determining methodology, 5) planning resources in context, lastly, step 6) finalising the plan in consideration with relevant stakeholders.

**Results:**

Quantitative data were collected from 32,785 antenatal and postnatal visits at the primary health care level, from 66 community engagement sessions, training assessments of community health workers, and standard health facility questionnaires. Forty-three focus group discussions, 38 in-depth interviews, and 23 structured observations were conducted to capture qualitative data. A total of 103 community engagement reports and 182 suspected pre-eclampsia case reports were purposively collected. Timing of data collection was staggered to understand feedback mechanisms that may have resulted from the delivery of the intervention. Data will be analysed using R and NVivo. Diffusions of innovations and realist evaluation theories will underpin analysis of the interaction between context, mechanisms and outcomes.

**Conclusion:**

This comprehensive approach can serve as a guide for researchers and policy makers to plan the evaluation of similar complex health interventions in resource-constrained settings, and to aid in measuring 'effectiveness' of interventions and not just 'efficacy'.

**Trial registration:**

This research is a part of the Community Level Interventions for Pre-eclampsia Study, NCT01911494. The trial is registered in Clinicaltrials.gov, the URL is https://clinicaltrials.gov/ct2/show/NCT01911494 The trial was registered on June 28, 2013 and the first participant was enrolled for intervention on March 1, 2014.

**Electronic supplementary material:**

The online version of this article (doi:10.1186/s12913-017-2124-4) contains supplementary material, which is available to authorized users.

## Background

The United Nations’ Millennium Development Goal (MDG) 5A called on countries for a 75% reduction in maternal mortality from 1990 to 2015. Nigeria has made progress in response to MDG 5A [1, 2], having achieved an apparent 39.7% reduction in the maternal mortality ratio (MMR) between 1990 and 2015 (comparable MMR of 1350 [UI 893–1820] deaths per 100,000 livebirths in 1990 and 814 [UI 596-1180] deaths per 100,000 livebirths in 2015); however, the World Health Organization (WHO) reports that there is a 10% chance that almost no maternal mortality reduction has occurred during this time [1].

Thaddeus and Maine proposed the ‘three delays model’ to explain how maternal mortality in low-and-middle-income countries may be addressed – namely, by addressing delays in: (i) seeking care, (ii) obtaining transport, and (iii) receiving appropriate care [3]. The Community Level Interventions for Pre-eclampsia (CLIP) Trial (NCT identifier: NCT01911494) aims to address these delays, and to reduce maternal and neonatal morbidity and mortality [4]. The CLIP intervention is ‘complex’ as it consists of five interrelated and interacting components: community engagement, community health worker strengthening, antenatal and postnatal monitoring, and, when indicated, community initiation of life-saving therapies to treat pre-eclampsia/eclampsia (ie. 10 g intramuscular magnesium sulfate with/without 750 mg methyldopa) in combination with urgent transport to an effective inpatient emergency obstetric care (EmOC) facility [4, 5]. The CLIP intervention multiple levels of the health system; for example, at the micro level, practitioner behavior is being modified, whereas at the macro-level, referrals are increased to secondary facilities. The interaction between the intervention and system level factors can inform how similar effects may be achieved across new contexts [6, 7].

Randomized control trials (RCTs) are considered the ‘gold standard’ for determining the efficacy of an intervention; however, effect sizes alone do not ensure that the measured effects of core components of a given intervention are generalizable [5]. Process evaluations aim to understand and assess the way in which an intervention is delivered. Furthermore, process evaluations can facilitate the answering of two pressing questions about complex interventions: firstly, what are the ‘active’ ingredients of the intervention; and secondly, what are the emerging adaptations of the intervention through its interaction with recipients [8, 9], who may be influenced by pre-existing circumstances, organisational norms, resources and attitudes [6–10]. Although theoretical evidence has emerged over the last decade on the approaches to process evaluation, there is a paucity of evidence specific to community-based trials, especially in resource-constrained settings. This evaluation plan can offer insight for policy and programmatic decisions. In this paper, we address this gap in knowledge by describing six stages of development of a process evaluation of a complex community-based maternal health intervention applied in a cluster randomised controlled trial. Existing methodologies are unable to asses the heterogeneous manner of the implementation of the CLIP intervention and are unable to provide a framework for assessment of mediating variables and contextual differences within different wards in each local government area (designated intervention cluster). An important feature in this methodology is its ability to not just identify complex causal pathways but to evaluate these mechanisms by analysing mediators. Furthermore, the construct of ‘adaptability’, i.e. tailoring of an intervention according to local needs and contexts, has been deliberately included to inform pragmatic contextual considerations for adaptation of the intervention. Adaptability is critical for informing real-world implementation.

## Methods

### Study setting

Five Local Government Areas (LGAs) in Ogun State, Nigeria were chosen to receive the CLIP intervention by stratified random sampling. The CLIP (Community Level Interventions for Pre-eclampsia) intervention was delivered as part of the CLIP cluster randomized pilot trial from March 2014 to May 2015 in two Local Government Areas, Yewa South and Remo North, and later expanded to an additional three Local Government Areas(I,e, Ijebu North East, Odeda, and Ogun Waterside) from May 2015 to January 2016 as part of the definitive CLIP cluster randomized trial. In Nigeria, the primary implementers of the intervention were the community health care providers- community health extension workers (CHEWs), health assistants (HAs) and staff nurses. The process evaluation protocol covers data gathered during the Feasibility Study (2013–2014) [11], and during delivery of the CLIP intervention (2014–2016).

The Research ethics boards at UBC Children's and Women's Health Centre of British Columbia and Olabisi Obabanjo University Teaching Hospital in Sagamu Nigeria provided ethical approval for the CLIP Cluster Randomized Controlled Trial (Number: H12-03497).

### Step-wise approach

The methods used to develop this process evaluation were adapted from Saunders et al [12] and tailored to the CLIP intervention in accordance with the Medical Research Council guidance [5, 8, 13]. Six steps were undertaken to: (i) to describe the intervention using a logic model to represent intervention activities, intended outcomes, theoretical constructs, and mediating factors [12]; (ii) to define complete and acceptable delivery of the intervention, in order to understand how the intervention may interact with the external MRC framework of process evaluation [5–9, 7, 13–17]; (iii) to develop process evaluation questions (iv) consider the relevant program resources (v) develop data management strategies (i.e., data sources, timing, and planning of data collection tools) using mixed-methods to answer the questions outlined in Step iii [10]; and (vi) to finalise the evaluation plan within an interdisciplinary team in collaboration with relevant stakeholders [9].

### Analysis plan

Quantitative data (such as that obtained using the PIERS on the Move mHealth platform), trial logs to monitor delivery of the intervention (community engagement logs, staff training logs, pre-post test questionnaires, drugs and devices tracking logs), observations checklists, budgets, and facility assessment data) will be analysed using simple descriptive analyses using Microsoft Access or R. Qualitative data (focus group discussions, key informant interviews, non-participant observations, pre-eclampsia case reports, community engagement field reports) will be analysed using thematic analysis in NVivo qualitative software. The use of established social theories is widely encouraged for process evaluations to allow for comparisons [8, 9, 18]. Therefore, building upon the Nigerian CLIP Feasibility Study [11], an adaptation of the diffusion of innovation theory [18, 19] was used to assess the CLIP intervention interacts with the system antecedents (context) to diffuse with the system (health system and community) for adoption by users (health workers and participants who receive the intervention). Realist theory was used to expand on the interaction between context and mechanisms to analyse identified mechanisms of action. The interaction of how ‘mechanisms’ and ‘context’ interact to produce ‘outcomes’ is represented in Fig. 2. Adaptability will be assessed to evaluate pragmatic contextual factors with delivery of implementation.

## Results

The six-step methodology was implemented in the context of CLIP intervention in Ogun State. At the time of writing, data collection was complete and plans for analysis are underway.

### Step 1: describe the components of the complex intervention

**Fig. 1:**
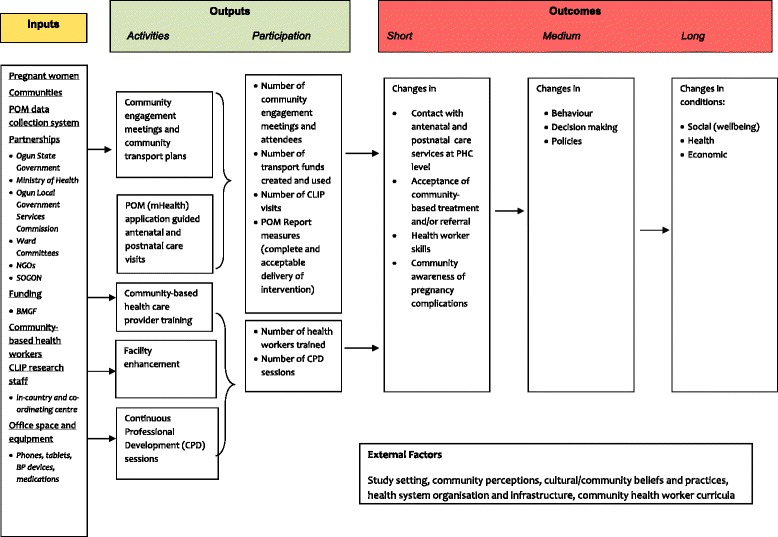
Logic model of the CLIP intervention in Ogun, Nigeria

The CLIP intervention consisted of five interrelated components as outlined in Additional file 1: Figure S1) community engagement; 2) staff and their preparation for CLIP; 3) mHealth tool and blood pressure measurement; 4) lowering severe hypertension using oral antihypertensives; 5) preventing & treating eclampsia using intramuscular MgSO4. Figure 1 presents the logic model developed to describe inputs, outputs (activities, participation), and their links to outcomes (short, medium and long term). These outcomes were developed with an iterative process in discussion with the local team, using knowledge of the participant’s planned participation and interaction with external factors. This logic model is used to identify the causal relationships between the different elements of the intervention.

### Step 2: constructs for evaluation

**Fig. 2:**
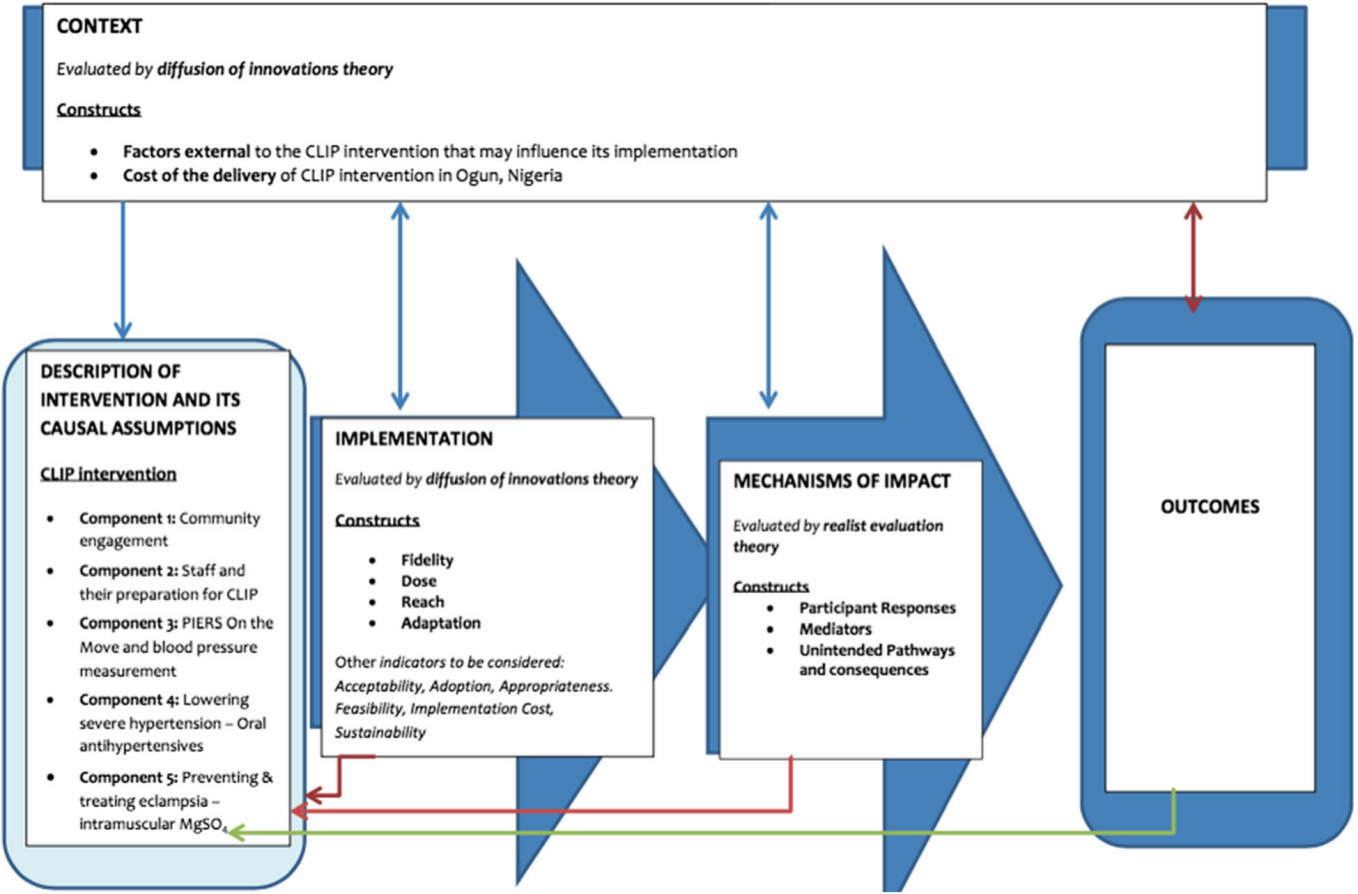
Constructs of process evaluation for the CLIP intervention in Ogun State: The key functions assessed will be implementation (the infrastructure through which intervention is delivered, how it is delivered and the ‘what’ ‘quantity and quality’ of intervention), mechanisms of impact (how interaction between intervention activities and participants effect outcomes), and context (evaluating external factors which shape or may be shaped by intervention). As evident, these functions are non-linear and mutually-informative

The second step is the identification of the dimensions for evaluation. The UK MRC guidance was used to determine relevant constructs, to generate evaluation questions, and define indicators for evaluation. Three dimensions, namely implementation, mechanisms of impact and context are being evaluated. Implementation represented the resources and processes through which the intervention was delivered, and the quantity and quality of delivery, utilizing indicators such as fidelity, reach, dose, adaptation, acceptability, feasibility, and appropriateness. Evaluating mechanisms of impact focussed on how intervention activities, and participants’ interactions with them, triggered change, using constructs such as participants’ responses, mediators, unintended consequences, and measurable indicators. Evaluation of the context examined how external factors influenced the delivery and functioning of the intervention. The interaction of these constructs is illustrated in Fig. 2.

### Step 3: develop a list of potential process evaluation questions

Through a combination of Steps 1 and 2, and through consideration of uncertainties of process interactions and of the causal assumptions underlying the mechanisms of intervention, the most important questions were systematically developed. Table 1 lists possible questions.

**Table 1:** Process evaluation questions for assessing implementation of intervention^a^

Constructs	Aims to be evaluated	Questions
Fidelity	1.To what extent was the CLIP intervention implemented consistently with the CLIP protocol? 2.To what extent was training provided as planned?	1.What constitutes as adherence for each component of the CLIP intervention? 2.What constitutes as competence for each component of the CLIP intervention?
Dose (delivered and received)	How much of the CLIP intervention was delivered? (‘dose’)	1.How many antenatal visits were delivered? 2.How many postnatal visits were delivered? 3.How many women received treatment (by MgSO4 and/or methyldopa) as per the recommendation? 4.How many women were referred to a higher level facility? 5.How many community engagement sessions were delivered to stakeholders?
	How much of the CLIP intervention was received? (‘dose received’)	1.How can participant behaviours be used to assess engagement? 2.With what specific aspects of the intervention (e.g., CLIP visits, community engagement sessions.) indicate assess participant reaction or satisfaction? 3.What are the expected follow-up behaviours: following recommendations generated in the POM mHealth tool, self-seeking regular antenatal/postnatal visits?
Reach	1.What is the reach of the CLIP intervention? 2.How are stakeholders and the community engaged in the process?	1.What are the target populations for each component of the CLIP intervention?
Adaptation	1.What were the alterations made to the CLIP intervention to adapt it to the Nigerian context?	1.How will the adaptations made in the design or delivery of the CLIP intervention be identified, tracked and monitored?
Context	1.What are the factors external to the CLIP intervention that may influence its implementation? 2.What is the cost of the CLIP intervention in Nigeria?	1.How will the contextual factors (including community, health system, sociodemographic, political factors) be identified, assessed and monitored?

^a^(adapted from Saunders et al [10])

The following research questions were considered:

Assessing implementation

1. Was the CLIP intervention implemented as planned? (‘fidelity’)
2. What was the extent to which pregnant women came into contact with the intervention, and the extent to which its delivery was of sufficient quality? (‘reach’)
3. How much of the CLIP intervention was delivered? (‘dose’)
4. What were the alterations made to the CLIP intervention to adapt it to the Nigerian context, and to achieve the intended protocol? (‘adaptation’)

Assessing mechanisms of impact

1. How did participants (community health care providers, pregnant women, families) interact with the intervention (‘participant responses’)?
2. What were intermediate processes which explain subsequent changes in outcomes? (‘mediators’)
3. What were unintended pathways or consequences of the intervention (‘unintended pathways and consequences)

Assessing context

1. What were the factors external to the CLIP intervention that may influence its implementation?
2. What was the cost of delivery of the CLIP intervention?

### Step 4: determine methods for evaluation

The methods for evaluation were both formative and summative; as a result, a mixed-methods approach was adopted. Qualitative methods generate hypotheses about causal mechanisms whereas quantitative methods can provide strong measures of fidelity, and test any emerging hypotheses.

### Sampling considerations

Process data was collected from a wide range of participants who were engaged with the intervention. Analysis will be undertaken for all collected data. In order to disentangle some specific elements of the intervention, we undertook purposive sampling. Sampling considerations were based on socio-demographic characteristics (for example: location of community engagement sessions) and organisational factors (for example: participants who were recommended treatment for suspected pre-eclampsia and eclampsia). Details on sampling consideration is provided in additional Additional file 2: Tables S1, Additional file 3: Table S2 and Additional file 4: Table S3.

### Timing of data collection

The interaction between participants and the intervention was dynamic; therefore, we collected data at different points in the study, including the Feasibility Study (2012–2013), and the Pilot/Definitive Study (May 2014- January 2016). The timing of data collection and data reporting were planned at different stages with the aim to provide iterative feedback (Tables 2 and 3). Evaluating at multiple time points highlighted how the intervention and context adapted to one another overtime.

**Table 2:** Considerations for planning CLIP Nigeria Process Evaluation Methods^a^

Methodological component	General definition	Relevance
Design	Timing of data collection	Pre-intervention and early-intervention
Data sources	Sampling and sources of data collection	1.Pregnant women receiving the CLIP intervention 2.Community health workers delivering the CLIP intervention at the primary care level (a small subset was sampled purposively) 3.Medical professionals receiving continuous professional development 4.Community engagement data
Data collection tools or measures	Instruments, tools, and guides used for gathering process- evaluation data	1.PIERS On the Move (POM) mHealth tool 2.Community engagement logs 3.Training pre-tests and post tests 4.Interviews 5.Focus group discussions 6.Case reports 7.Device purchase and tracking logs 8.Study budget
Data collection procedures	Protocols for how the data collection tools are administered	1.Data collection protocol 2.Training manuals
Data management	Procedures for gathering data from the field and data entry; data quality checks; data security; data monitoring procedures.	1.Data security management, such as encryption of POM data using Blowfish algorithm, and creation of audit trails for new records 2.Regular data monitoring reports 3.Field activity logs: community engagement, trainings 4.Data monitoring plan
Data analysis and synthesis	Statistical and/or qualitative methods used to analyze, synthesize, and/or summarize data.	1.Thematic analysis 2.Descriptive statistics using R analysis program 3.Framework analysis

^a^(adapted from Saunders et al [10])

**Table 3:** Sources and data collection tools

Source		Data collection tool	Timing of data collection
Pregnant women registered in the CLIP Study	Registered women who receive the CLIP intervention at the primary health centres	PIERS On the Move (POM) application	During intervention
	Registered women who received recommendations from the intervention	Case reports	During intervention
	Women who received community engagement sessions	Community engagement logs and reports	During intervention
	Registered women who are a part of leading community engagement efforts (survivors clubs)	Community engagement logs	During intervention
Community health care providers	CHEWs/HA’s who receive CLIP training	• Pre- and post- tests • Structured observations • One on one interviews	Pre-intervention and during intervention
	Medical Officers	Focus group discussions	Pre-intervention
	Medical professionals who participated in CPD (Continuous Professional Development) events	CPD activities Log	Pre-intervention, and during intervention
Community members/stakeholders	Community members who participated in community engagements	Community engagement logs	During intervention
	State government officials	Meeting reports/event reports	Pre-intervention, during intervention and during-intervention
	Local Government officials	• Interviews • Meeting reports/event reports	Pre-intervention, during intervention and during-intervention
	Grassroots leaders	• Interviews • Meeting reports/event reports	Pre-intervention, during intervention
	Transport unions	Community engagement logs	During intervention
	Shopkeepers	Community engagement logs	During intervention
Research Staff		Key informant interviews	Pre and post- intervention

### Data sources and data-collection tools

1. *Implementation fidelity:* Data sources included PIERS On the Move (POM) mHealth platform reports used by community health workers as a decision aid for triage and treatment, reports from community mobilisers and observations of community health workers when providing the intervention.
2. *Dose (delivered and received):* Data sources included PIERS On the Move (POM) mHealth platform, and case reports generated by community health care workers.
3. *Reach:* Data sources include PIERS On the Move (POM) mHealth platform, community engagement logs and case reports.
4. *Adaptation:* Data sources included key informant interviews with the study team, community engagement reports, community health care training records, log of updates to the Piers On the Move application and related training records.

*Context:* Data sources included focus group discussions, in-depth interviews conducted with a variety of stakeholders during the CLIP Feasibility Study [11], and intervention costing data to assess barriers and facilitators to implementation.

### Qualitative methods

#### Focus group discussions

Forty-one focus group discussions were conducted in the CLIP Feasibility Study, before delivery of the intervention. Focus groups were held with representatives of the health system and the community to explore issues such as feasibility and acceptability of the intervention. In addition, two focus groups were held with community health care workers during delivery of the intervention in local language. This second set of focus groups was used to assess the competency and experience of health workers with the Microlife VSA blood pressure device [20].

### In-depth interviews

Thirty-eight in-depth interviews were conducted to gain an understanding of complex and sensitive issues associated with implementation of the intervention, such as those related to task-shifting at the community level. Key informant interviews were held with field staff and stakeholders to explore the interaction of the] delivery of the intervention and the context.

### Non-participant observations

Field notes were taken during structured observations at primary health centers prior to implementation of the intervention. The observations were conducted to describe the setting prior to the intervention. In addition, six non-participant observation sessions of community health workers undertaking seventeen blood pressure monitoring activities were completed.

### Community engagement reports

One hundred and three reports from two hundred and ninety-one community engagement sessions were collected and reviewed to capture the responsiveness of the communities to the intervention. Responsiveness refers to the community’s participation in the implementation and uptake of the intervention, which could be an important mediating factor. These detailed report highlighted perceived value of the intervention by the communities, as well as challenges in the delivery of the intervention.

### Pre-eclampsia case reports

One hundred and eighty one case reports were collected for all patients who were recommended treatment in accordance with the CLIP intervention. These written reports were documented by a surveillance officer and included demographic information, treatment and referral details, and follow-up visits was recorded for all participants. In addition to the clinical details, which were captured on the POM application, these case reports allowed researchers to capture the ‘spirit’ of the intervention. By documenting the complete medical journey of the patient, these case reports provide direct understanding of task-shifting to community health care providers, and larger effects on the health system [8].

### Quantitative methods

#### PIERS on the move data

The POM mHealth application [21, 22] was used to guide care and capture critical clinical data related to delivery and acceptance of the intervention. 32,882 antenatal and postnatal visits were recorded electronically and synchronized regularly with the central database in Nigeria. Data collected through the POM application included demographics, clinical signs and symptoms. Along with serving as a decision aid and capturing electronic data, the adaptations made to the POM application via iterative feedback from the field provided robust information on the implementation challenges of the intervention. Regular data monitoring, cleaning, and reporting allowed for real time feedback to the field team, as well as safety monitoring of participants. Data captured using the POM device is described in Additional file 5: Figure S2.

### Community health worker pre- and post-test assessments

Pre- and post-test assessments were conducted during health worker trainings. The results of these tests were used to evaluate the fidelity (was the training received as planned), dose (how much training was received), reach (how many health workers were trained), and adaptation (how was CLIP training integrated into health system curriculum) of the intervention.

### Step 5: consider program resources and context

After the formulation of methods in Step 4, this next stage considers available resources to answer the questions outlined in Step 3 [10]. The pre-intervention data collected in the CLIP Feasibility Study identified the appropriate allocation of resources necessary to answer questions for process evaluation, such from facility assessment to evaluate health system infrastructure, and evaluation of stakeholders’ commitment to task-shifting. Furthermore, the availability of trained health workers to deliver the intervention, and trained community mobilisers for engaging communities, along with local research staff to monitor data collection was assessed.. In addition, the research study team was sensitized to the theories of process evaluation, as it requires an investment by those with a strong working knowledge of relevant theoretical perspectives [8].

### Step 6: finalise the process evaluation plan

Using the formative feedback generated in Steps 3 to 5, the final process evaluation plan was conceptualized. The comprehensive plan is displayed in additional files 2, 3 and 4 (Table S1, Table S2 and Table S3).

## Discussion

There is no standard method to process evaluations, owing to the difference ins the design of the interventions. We propose step by step guidance to evaluate generalizability for community based intervention which is grounded in validated social theories. Complex interventions are often described as a ‘black box,’ as the ‘active’ ingredients are unknown, as well as which processes mediate or influence outcomes (causal mechanisms) [5, 8, 9]. In addition to the challenge in disentangling the effects of the components of a complex intervention, emerging outcomes may be non-linear (i.e., unintended outcomes resulting from indirect interactions [5]. The context may influence how the intervention is delivered; nevertheless, the theory of ‘mutual adaptation’ indicates that the context may also change in response to the intervention [23, 24]. The intervention allows for the study of reciprocal interactions between context and intervention [19]; for instance, women in this study determined the number of antenatal and postnatal visits (‘dose’ of the intervention) delivered by way of their care-seeking practices to the primary health center. The exploration of such reciprocal interaction in the cause and effect pathway can provide insight into unanticipated mechanisms [5, 8, 9, 24].

There is no standard method for the process evaluation of complex interventions. A critique of existing process evaluation approaches is that they do not empirically test causal mechanisms [8, 25]; however, the methodology presented here allows us to test these causal assumptions through mediation analysis. In this context, mediation analysis is done by testing assumptions stated in Steps 1 and 2. The methodology allows us to understand how ‘X’ interacts with a mediating variable ‘Y’ to produce outcome ‘Z’. For instance, when intervention ‘X’ is implemented (community engagement in CLIP), it leads to change in mediating variable ‘Y’ (knowledge awareness and birth preparedness) which produces the outcome ‘Z’ (increase in antenatal care visits sought at the PHC). The emphasis on mechanisms of change in this evaluation, such as examining immediate benefits (for example, increased provider knowledge) rather than relying on long-term health outcomes, aids in planning of similar health interventions. The methodology has comprehensive and flexible indicators that can be replicated to ensure comparability between studies of cluster RCTs. In this paper, we build on process evaluation guidance using established social theories, such as those by Steckler and Linnan [14], Greenlagh et al [19], Blackwood et al. [26] and methodologies proposed by the UK MRC guidance [25].

While the methodology described in this paper may offer robust measures of the process indicators, external validity of conclusions about effectiveness can best be complemented by efficacy studies using a RCT. The methodology allows to examine the internal validity of the efficacy of the intervention by assessing the implementation (quantity and quality) of what is delivered. The face validity and criterion validity of the methodology will be tested by the degree of convergence or divergence of the constructs to their hypothesised function in the CLIP Trial (Additional file 2: Tables S1, Additional file 3: Table S2 and Additional file 4: Table S3).

Furthermore, when planning evaluations, it is crucial to distinguish between the intent to determine ‘effectiveness’ versus ‘efficacy’. This decision may be informed by the context (open vs. closed) and the location of intervention delivery [23, 26, 27]. RCTs are best suited to evaluations of ‘efficacy’ in a ‘closed’ system, whereby all other factors are equal, differences in the delivery of the intervention to the intervention arm allows for a valid assessment of the hypothesised health outcome [23, 26]. On the contrary, in an ‘open’ system, as was the case in this study, it is more appropriate to measure the ‘effectiveness’ of the intervention by examining factors related to implementation, and the mechanisms by which organisational structures, behaviour, and cultural norms impact delivery and uptake of the intervention. The ‘efficacy’ of the CLIP intervention will be assessed from the results of three RCTs in India, Pakistan and Mozambique [4]. Efficacy will be determined based on the ability to demonstrate a reduction of maternal and perinatal morbidity and mortality.

This evaluation design is strengthened by the use of established social theories, allowing for comparisons between studies [6, 18, 19]. The use of mixed methods and design allows for reciprocal interactions between context and intervention [19].

Furthermore, integration of realist theory in analysis will help identify the likelihood of outcomes related to the context [6, 26, 28]. By placing mechanisms at their core, realist evaluation theories can uncover which intervention activities work, for whom and under what context [9, 6].

The identification of core components of the intervention can inform wider implementation. The design of this evaluation is process- rather than package-oriented, and considers the possible effects across all major sections of the health system. The ecological nature of this evaluation allows identification and generation of new mechanisms of action. In the dynamic context of less-developed countries, where competing priorities from development partners and parallel vertical programs can create unintended outcomes, an evaluation of this nature can guide strategic investments in health.

## Conclusion

The methodology outlined here can be used to develop similar process evaluations of complex health interventions for community- based studies of maternal and perinatal health. There are inherent limitations and strengths to this methodology for evaluation of complex health care interventions. Although the extent to which effectiveness can be determined with hard predictive probability may be curtailed, this adapted methodology provides a step-wise approach to develop plausible explanations for causal mechanisms, and interaction of a complex intervention in cluster randomized control trials.

## Additional files

Additional file 1: Figure S1. Components of the CLIP intervention. (PDF 67 kb)

Additional file 2: Table S1. Final plan to measure the construct of implementation. (DOCX 17 kb)

Additional file 3: Table S2. Final plan to assess the mechanisms of impact of CLIP intervention. (DOCX 15 kb)

Additional file 4: Table S3. Final plan to assess context of CLIP intervention. (DOCX 16 kb)

Additional file 5: Figure S2. Information captured via the POM app in Ogun, Nigeria. (PDF 30 kb)

## Abbreviations

CE: Community engagement
cHCP: Community health care provider
CHEW: Community health extension worker
CLIP: Community level interventions for pre-eclampsia
CPD: Continuous professional development
FGD: Focus group discussion
HA: Health assistant
IDI: In-Depth interview
MDG: Millennium development goal
MMR: Maternal mortality ratio
MRC: Medical research council
POM: PIERS on the move
RCT: Randomised controlled trial
SDG: Sustainable development goal
SOGON: Society of obstetricians and gynaecologists of Nigeria
