# Supplementary material for: A process evaluation plan for assessing a complex community-based maternal health intervention in Ogun State, Nigeria
Source: BMC Health Serv Res. 2017 Mar 28;17:238. doi: 10.1186/s12913-017-2124-4 (PMC5371276; doi:10.1186/s12913-017-2124-4)
Supplement: Supplementary file 1 — Components of the CLIP intervention. (PDF 67 kb) [file 12913_2017_2124_MOESM1_ESM.pdf]

Component 1: Community engagement

Engagement with pregnant women, their families and communities was undertaken to deliver key health messages: awareness about hypertension in pregnancy, warning signs and symptoms, and the CLIP intervention. An essential component of community engagement is feedback about the 'great saves' and 'near misses'. In some regions in Nigeria, women who were diagnosed, triaged and treated as a result of the CLIP intervention created local 'survivors clubs'. This grassroots movement served to educate the communities about the importance of timely action. Stakeholders in the community include transport union leaders, barbers, and mothers-in-law, husbands, and local leaders. Furthermore, community engagement encompasses those in the local government service commission, medical officers, and state government officials.

Component 2: Staff and their preparation for CLIP

In Nigeria, Community Health Extension Workers and Health Assistants are responsible for primary health care. The training programme for the health workers were developed after an extensive review of both the standard training programme and the additional requirements needed for the intervention. In addition, training in the care of the hypertensive disorders of pregnancy was offered to all referral centres, and hospital based-medical and nursing staff across all public and private sector rural health centres.

Component 3: PIERS On the Move and blood pressure measurement

The miniPIERS model (Pre-eclampsia Eclampsia Integrated Assessment of RiSk) based on a combination of maternal and fetal predictors (purely symptom- and sign-based) accurately identifies women at incremental risk of maternal complications of pre-eclampsia (4). This model is integrated into a mHealth platform that generates recommendations for triage and treatment. Blood pressure is measured using the automated sphygmomanometers, validated for use in LMIC settings, the Microlife BP 3AS1-2 and the Microlife CRADLE VSA, developed for Professor Shennan's parallel CRADLE (Community blood pressure monitoring in Rural Africa: Detection of underLying pre-Eclampsia) research programme (28).

Community health care providers were trained to assess pregnant women at least four times antenatally and at least once postnatal. During these visits, they enquire about women's symptoms, assess gestational age, take blood pressure, and check for proteinuria. The recommendations resulting from the POM tool aid in the appropriate treatment and transport actions (18). *
from May 2015-Jan 2016

Component 4: Lowering severe hypertension – Oral antihypertensives

750mg (alpha-) methyldopa is provided as a safe and effective oral agent in the community for hypertension in pregnancy (4)

Component 5: Preventing & treating eclampsia – intramuscular $MgSO_4$

A single dose of 10g of MgSO4 is delivered intramuscularly.; MgSO4 is the agent of choice for the prevention and treatment of eclampsia.
