# Supplementary material for: A process evaluation plan for assessing a complex community-based maternal health intervention in Ogun State, Nigeria
Source: BMC Health Serv Res. 2017 Mar 28;17:238. doi: 10.1186/s12913-017-2124-4 (PMC5371276; doi:10.1186/s12913-017-2124-4)
Supplement: Supplementary file 2 — Final plan to measure the construct of implementation. (DOCX 17 kb) [file 12913_2017_2124_MOESM2_ESM.docx]

**Table S1: Final plan to measure the construct of implementation**

| **Construct** | **Evaluation question** | **Proposed indicator** | **Source** | **Sample size** | **Time of data collection** |
| --- | --- | --- | --- | --- | --- |
| Fidelity | Is the CLIP intervention being implemented as outlined in the protocol? | Number of Community engagement (CE) meetings per cluster | CE logs | All CE meetings | During intervention |
|  |  | Number of stakeholder groups represented at CE meetings | CE logs | All CE meetings | During intervention |
|  |  | Number of community health care provider (cHCP) training sessions | Health worker training logs | All trainings | At completion of cHCP trainings |
|  |  | cHCP Training content | CHEW/HA training materials | All CHEW/HA trainings | At completion of CHEW/HA trainings |
|  |  | Number of women with a POM visit every 4 weeks | POM report | All POM visits | During intervention |
|  |  | Percentage of women accepting recommendations (overall acceptance rate) | POM report | All POM visits | During intervention |
|  |  | Number of Continuous Professional Development (CPD) activities | CPD logs | All CPD events | Pre- and during intervention |
|  |  | Number of Facility enhancement items distributed (*ampoules of MgSO_4_, BP devices, boxes of Aldomet)* | Facility enhancement coordinators | All facility enhancement activities | Pre-, during and post- intervention |
| Reach | What is the reach of the CLIP intervention?  How are stakeholders and the community engaged in the process? | Cluster areas with one/more CE meetings | CE logs | All CE meetings | During intervention |
|  |  | Number of women with four antepartum and atleast one postpartum POM visits | POM report | All POM visits | During intervention |
|  |  | Number of blood pressure machines and devices^⌂^ purchased | Device purchase orders | All devices | Post intervention |
|  |  | Number devices distributed to community | Devices Tracking log | All devices | Post intervention |
|  |  | Number of devices distributed to facility | Devices Tracking log | All devices | Post intervention |
|  |  | Numbers of devices remaining in functional condition | Devices Tracking log | All devices | Post-intervention |
| Dose | What is the ‘dose’ of the CLIP intervention? | Number of CE meetings | CE logs | All CE meetings | During intervention |
|  |  | Number of CE topics covered per meeting held | CE logs | All CE meetings | During intervention |
|  |  | Number and types of community groups created | Community supervisors | All CE meetings | During and post-intervention |
|  |  | Number of antepartum and postpartum visits with identification of severe and non-severe hypertension | POM report | All POM visits | During intervention |
|  |  | Number of visits with recommendations accepted for treatment and transport | POM report | POM visits with a transport recommendation | During intervention |
| Adaptations | What are the alterations made to the CLIP intervention to meet the context? | Description of all new community groups created to engage communities | CLIP Research Staff | All community activity during the project | During intervention |
|  |  | PHC-based CLIP visits due to human resource constraints | CLIP Feasibility Report  CLIP Trial Protocol | Not applicable | During intervention |
|  |  | Use of Health assistants in CLIP given their work in private institutions | CLIP Trial Protocol | Not applicable | During intervention |
|  |  | New Blood pressure device to evaluate shock index | CLIP Trial Protocol | Not applicable | During intervention |
|  |  | Updates to the POM mHealth application | CLIP Trial Protocol | Not applicable | During intervention |
