# Supplementary material for: A process evaluation plan for assessing a complex community-based maternal health intervention in Ogun State, Nigeria
Source: BMC Health Serv Res. 2017 Mar 28;17:238. doi: 10.1186/s12913-017-2124-4 (PMC5371276; doi:10.1186/s12913-017-2124-4)
Supplement: Supplementary file 3 — Final plan to assess the mechanisms of impact of CLIP intervention. (DOCX 15 kb) [file 12913_2017_2124_MOESM3_ESM.docx]

**Table S2: Final plan to assess the mechanisms of impact of CLIP intervention**

| **Construct** | **Evaluation question** | **Proposed indicator** | **Source** | **Sample size** | **Time of data**  **collection** |
| --- | --- | --- | --- | --- | --- |
| Participant responses | How do participants interact with a complex intervention? | Reported self-efficacy of health workers | Focus Group Discussions | Two Focus Group Discussions | During intervention |
|  |  | Pre- and post- intervention antenatal care day observation | Observation record/field notes | Four PHCs at each time point | Pre- and post-intervention |
|  |  | Pre- and post- test for training of CHEWs and HA’s | Skills and knowledge assessment | Number of training sessions | Pre-intervention |
|  |  | Health worker case studies | Case study reports | Visits with referral recommendation | During intervention |
| Mediators | What are intermediate processes which explain subsequent changes in outcomes? | Community support for the CLIP intervention | CLIP Research staff  Community engagement log  Feasibility report | Not applicable | Pre- intervention and during intervention |
|  |  | Women without follow up visits following recommendation in previous antenatal/postnatal visit | POM report | Visits with referral recommendation | During intervention |
|  |  | Perspectives of Medical officers on CLIP-related task-shifting activities | Interviews |  | Pre- intervention |
| Unintended pathways and consequences | What are unintended pathways or consequences of the intervention? | Number of total adverse events | POM report | Visits with any recommendation | During intervention |
|  |  | Number of cases of infection at injection site due to MgSO4 administration in community | POM report | Visits with MgSO_4_ recommendation | During intervention |
|  |  | Number of hematomas due to MgSO4 administration in community | POM report | Visits with MgSO_4_ recommendation | During intervention |
|  |  | Number of transport related injuries | POM report | visits with referral recommendation | During intervention |
