# Supplementary material for: A process evaluation plan for assessing a complex community-based maternal health intervention in Ogun State, Nigeria
Source: BMC Health Serv Res. 2017 Mar 28;17:238. doi: 10.1186/s12913-017-2124-4 (PMC5371276; doi:10.1186/s12913-017-2124-4)
Supplement: Supplementary file 4 — Final plan to assess context of CLIP intervention. (DOCX 16 kb) [file 12913_2017_2124_MOESM4_ESM.docx]

**Table S3: Final plan to assess context of CLIP intervention**

| **Construct** | **Evaluation question** | **Indicator** | **Source** | **Sample size** | **Time of data collection** |
| --- | --- | --- | --- | --- | --- |
| Context | What are the factors external to the CLIP intervention that may influence its implementation? | Study setting | - Manuscripts - Feasibility report | Feasibility Study | Pre-intervention |
|  |  | Barriers to CLIP | Feasibility report | Feasibility Study | Pre-intervention |
|  |  | Facilitators to CLIP | Feasibility report | Feasibility Study | Pre-intervention |
|  |  | Community perceptions | Feasibility report | Feasibility Study | Pre-intervention |
|  |  | Health facility infrastructure | Feasibility report | Feasibility Study | Pre-intervention |
|  |  | Community health care provider (cHCP) curricula | Feasibility report | Feasibility Study | Pre-intervention |
|  |  | Demographic information of CLIP women | POM Report |  | During intervention |
|  | What is the cost of delivery of the CLIP intervention in Nigeria? | Cost of drugs and devices (MgSO_4_, Aldomet, training, BP devices, mHealth platform etc.) | CLIP study budget | Not applicable | Pre-, during and post-intervention |
|  |  | Costs related to referral and emergency obstetric transport | Transport log | Not applicable | Post-intervention |
