# Supplementary material for: A process evaluation plan for assessing a complex community-based maternal health intervention in Ogun State, Nigeria
Source: BMC Health Serv Res. 2017 Mar 28;17:238. doi: 10.1186/s12913-017-2124-4 (PMC5371276; doi:10.1186/s12913-017-2124-4)
Supplement: Supplementary file 5 — Information captured via the POM app in Ogun, Nigeria. (PDF 30 kb) [file 12913_2017_2124_MOESM5_ESM.pdf]

- Patient information
- Contact details
- Initial assessment (emergency conditions)
- Pregnancy status
- Fetal status
- Date of delivery
- Blood pressure
- Urinary protein (on first visits and if hypertensive)
- Estimated date of delivery
- Symptoms (if hypertensive)
- Recommendations (generated by the POM application)
- Follow-up
- Adverse events (hematoma. infection at injection site and injury related to transport
